# Electrospun Fibrous Nanocomposite Sensing Materials for Monitoring Biomarkers in Exhaled Breath

**DOI:** 10.3390/polym15081833

**Published:** 2023-04-10

**Authors:** Yin-Hsuan Chang, Ting-Hung Hsieh, Kai-Chi Hsiao, Ting-Han Lin, Kai-Hsiang Hsu, Ming-Chung Wu

**Affiliations:** 1 Department of Chemical and Materials Engineering, Chang Gung University, Taoyuan 33302, Taiwan; 2 Division of Pediatric Neonatology, Department of Pediatrics, Chang Gung Memorial Hospital, Linkou, Taoyuan 33305, Taiwan; 3 Green Technology Research Center, Chang Gung University, Taoyuan 33302, Taiwan

**Keywords:** electrospinning, exhaled breath sensors, diabetes, acetone sensor

## Abstract

Human−exhaled breath mainly contains water, oxygen, carbon dioxide, and endogenous gases closely related to human metabolism. The linear relationship between breath acetone and blood glucose concentration has been revealed when monitoring diabetes patients. Considerable attention has been directed toward developing a highly sensitive volatile organic compounds (VOCs) sensing material that can detect breath acetone. In this study, we propose a tungsten oxide/tin oxide/silver/poly (methyl methacrylate) (WO_3_/SnO_2_/Ag/PMMA) sensing material fabricated using the electrospinning technique. By monitoring the evolution of sensing materials’ extinction spectra, low concentrations of acetone vapor can be detected. Moreover, the interfaces between SnO_2_ and WO_3_ nanocrystals construct n−n junctions, which generate more electron–hole pairs than those without such structure when the light strikes. This helps to improve the sensitivity of sensing materials when they are subjected to acetone surroundings. The established sensing materials (WO_3_/SnO_2_/Ag/PMMA) exhibit a sensing limit of 20 ppm for acetone vapor and show specificity for acetone even in ambient humidity.

## 1. Introduction

Diabetes is recognized as one of the fastest−growing diseases and is one of the top ten causes of death globally in the 21st century. Predictions suggest the number of diabetes patients worldwide will increase to 643 million by 2030 and 783 million by 2045 [1]. Diabetic ketoacidosis (DKA) is an acute metabolic complication in type 1 diabetes mellitus, which is due to the deficiency of insulin. Blood glucose level is an important clinical diagnosis for diabetes, which requires routine blood glucose monitoring to inform recommendations for adjustments related to diet, medication, and exercise regimens [2,3,4]. Besides, inadequate insulin results in the decomposition and oxidation of free fatty acids, leading to increased ketone in the blood [5,6]. Therefore, acetone in the breath can be seen as a significant biomarker for diabetes as an alternative to traditional finger−pricking to monitor blood glucose [7,8,9].

Human−exhaled breath mainly contains 78% nitrogen, 16% oxygen, 4% carbon dioxide, 0.09% argon and over 3500 volatile organic compounds (VOCs) [10,11,12]. After the inhaled air goes into the alveoli in the lungs, molecules pass the blood–air barrier, allowing the excretable metabolic products to be excreted in the exhaled breath. Therefore, the exhaled breath can represent the endogenous metabolic fingerprint [13,14]. For healthy people, breath acetone concentrations range from 0.3 ppm to 0.9 ppm. Diabetes can be identified once the breath acetone concentration exceeds 1.8 ppm. Owing to this relationship, developing a cost−effective and reliable exhaled breath sensing device is urgent. Currently, a variety of acetone detection methods have been reported, including colorimetric sensors [15,16], optical sensors [17,18], metal oxide sensors [8,19], monolayer−coated metal nanoparticle sensors [20], etc. Liao et al. demonstrated the detection of acetone using a luminescent chemosensor. When europium (Eu)−containing covalent organic framework (DhaTab−COF−EuIL) was exposed to acetone, it revealed significant luminescence quenching toward acetone caused by competitive absorption of the light source’s energy and led to the transfer of photoinduced energy [21]. In 2021, Wang et al. updated the possibility of using colorimetric sensors to track breath acetone accurately. The protons produced by the reaction between acetone and hydroxylamine sulfate induced the pH change and visualized the existence of acetone through color changes [22].

A wide variety of sensing materials, including conjugating polymers [23,24], metal–organic frameworks [25,26], and semiconductor materials [27,28] have been utilized for sensing volatile organic compounds (VOCs). Tungsten trioxide (WO_3_) has attracted considerable attention as a gas−sensing material due to its gasochromic properties, high stability, and high sensitivity. Likewise, tin oxide (SnO_2_), an n−type semiconducting metal oxide, has also been extensively reported as a sensing material. Li et al. utilized Ru−doped SnO_2_ for acetone gas sensing. The electron depletion layer formed at the p−n heterojunction resulted in more chemisorbed oxygen species and further improved the gas−sensing performance [29]. Among all reported nanoscale materials, the electrospun fiber with a large specific surface area has been widely used to develop as sensing materials. Qiu et al. demonstrated a conductive film for detecting vital bio−signals. The conductive film consisted of annealed electrospun phenolic resin fibers on graphene which is then semi−embedded into a styrene–ethylene–butylene–styrene (SEBS) elastomer. The high−temperature annealing process creates strong interactions between the polymer fibers and graphene, resulting in a network that mimics the structure of a bird’s nest [30]. The electrospinning method allows diversity in material selection and preparation methods. Solution properties, operating parameters, and environmental conditions are crucial factors in the morphology of electrospun nanofibers. Incorporating functional nanoparticles into electrospun fibers can expand the applicability of the materials.

In this study, we developed highly sensitive tungsten oxide/tin oxide/silver/poly (methyl methacrylate) (WO_3_/SnO_2_/Ag/PMMA) sensing materials to detect acetone in exhaled breath. It takes advantage of the distinct optical property of WO_3_, SnO_2_, and Ag surface plasmon resonance. The correlation between the sensitivity and UV−ozone etching treatment on the WO_3_/SnO_2_/Ag/PMMA sensing materials was investigated to understand the modulation of VOC sensitivity. By monitoring the evolution of sensing materials’ extinction spectra, low concentrations of acetone gas can be detected. It is hoped that the enhanced sensitivity and accuracy of sensing materials can be used to identify early−diabetic patients, reduce the waste of medical resources, and enhance the quality of life for patients.

## 2. Materials and Methods

### 2.1. Preparation of Freestanding WO_3_/SnO_2_/Ag/PMMA Sensing Materials

Silver nanoparticles (Ag NPs) were synthesized using solution−based chemical reduction routes as reported by Polavarapu et al. [31]. Silver nitrate (AgNO_3_, >99.5%, Sigma−Aldrich) was used as a precursor and oleylamine (C_18_H_37_N, >98%, Sigma−Aldrich) was used as a reducing agent. Ag NPs were obtained by dissolving 0.6 mmol of AgNO_3_ and 6.0 mmol of oleylamine in 50.0 mL of chlorobenzene (C_6_H_5_Cl, >99%, Acros) in a four−necked flask. The reaction mixture was kept at 120 °C for 1 h with continuous stirring under a nitrogen atmosphere and then cooled down to room temperature. The Ag NPs were dispersed in chlorobenzene and further materials during fabrication. SnO_2_ NPs were synthesized by hydrothermal method. An amount of 0.53 g of SnCl_4_·5H_2_O (>98.0%, Nacalai Tesque) was dissolved in 10 mL of benzyl alcohol (C_7_H_8_O, 99%, ACROS) with continuous stirring for 10 min. The mixture was transferred into a Teflon−lined autoclave and placed inside a 120 °C oven for 12 h. The resulting SnO_2_ NPs were centrifuged at 5000 rpm for 10 min and washed three times with diethyl ether. WO_3_ was synthesized by the solvent–thermal method. Tungsten chloride (WCl_6_, 99.9+%, ACROS) was used as the soluble tungsten source and ethanol was used as the solvent. WCl_6_ (50.0 mg) was added to 40.0 mL of ethanol (C_2_H_6_O, 95%, ACROS) and stirred for 30 min. The mixture was transferred into a Teflon−lined autoclave and placed in a 180 °C oil bath for 12 h. The resulting WO_3_ NPs were centrifuged at 5000 rpm for 15 min and washed three times with DI water.

To prepare WO_3_/SnO_2_/Ag/PMMA sensing materials, the electrospinning precursor solution was prepared by dissolving various ratios of 1.0 wt% Ag, 1.0 wt% SnO_2_, 1.5 wt% WO_3_, and 5.0 wt% PMMA in dimethylformamide (DMF, C_3_H_7_NO, >99.5%, Fisher Scientific). PMMA (MW∼120,000 Da) was purchased from Sigma−Aldrich and used without further purification. The WO_3_/SnO_2_/Ag/PMMA precursor solution was stirred at 80 °C for 3 h until the polymer was completely dissolved. Then, the WO_3_/SnO_2_/Ag/PMMA nanofibers were fabricated using the electrospinning technique from the precursor solution mentioned above. The electrospinning device includes a syringe pump (KDS−100, KD Scientific Inc., Holliston, MA, USA), a high−voltage power supply (SC−PME50, Cosmi Global Co. Ltd., New Taipei City, Taiwan), and a grounded rotary collector with a diameter of 15.0 cm and a length of 15.0 cm (FES−COS, Falco Tech Enterprise Co. Ltd., New Taipei City, Taiwan). The optimized electrospun parameters in the operation were an applied voltage of 10.0−25.0 kV, a working distance of 10.0 cm, a flow rate of 0.5 mL/h, a solution volume of 10 mL, and a receiving plate speed of 500 rpm. For the surface treatment of sensing materials, WO_3_/SnO_2_/Ag/PMMA hybrid fiber was subjected to a 10 min surface etching treatment by ultraviolet light/ozone etching technology (UV/Ozone, IAST0001−020, STAREK Scientific Co., Taipei City, Taiwan) to increase its surface area. The UV/Ozone was equipped with 4 UVC lamps (PL−L, 36W, λ max = 254 nm, Philips, Amsterdam, Netherlands).

### 2.2. Material Characterization

The microstructures of Ag NPs, SnO_2_ NPs, and WO_3_ NPs were observed by using the transmission electron microscope (TEM, JEM−2100Plus, JEOL, Tokyo, Japan). The percentage distribution of particles’ sizes was obtained from individual measurements of at least 100 particles. UV–vis absorption spectra of various nanoparticles were measured by a UV−VIS spectrophotometer (UV−1900i, Shimadzu, Kyoto, Japan). For the morphology of various sensing material, field−emission scanning electron microscope (FESEM, model SU8010, Hitachi, Tokyo, Japan) were used for the observation. At least 100 fibers were counted for obtaining fiber diameter distribution.

### 2.3. Optical Measurement of Extinction Spectrum

The VOCs were injected into a 4.5 cm by 4.0 cm by 4.0 cm quartz glass container to simulate the concentration of breath acetone. Measurements are taken at room temperature (25 °C) with a relative humidity range of 60%. To prevent the interference of environmental humidity, the response time is defined as extinction change value exceeding the extinction change maximum (∆Emax) of blank (air). Namely, as the ∆E that is over the threshold of the ∆E_max, air_. In the beginning, the WO_3_/SnO_2_/Ag/PMMA sensing material was placed in the middle of the container. The target solvent with a specific concentration was injected into the quartz chamber and the extinction spectrum was measured every 30 sec immediately. The extinction spectrum was measured by UV−VIS spectrophotometer from the wavelength 400 nm to 1000 nm. The maximum extinction change (ΔE) of the peak is determined as the sensitivity for a particular VOC. The absorption behavior of the sensing material results in a variety of extinction intensities. Therefore, the extinction change of the peak is defined by the extinction before and after the VOC sensing. The extinction formula is defined as follows

$$\Delta E=E_{t}-E_{t_{o}}$$

where $E_{t_{o}}$ represents the extinction intensity of the peak before the VOCs detection and $E_{t}$ represents the extinction intensity of the peak after the VOCs detection.

## 3. Results

**Figure 1:**
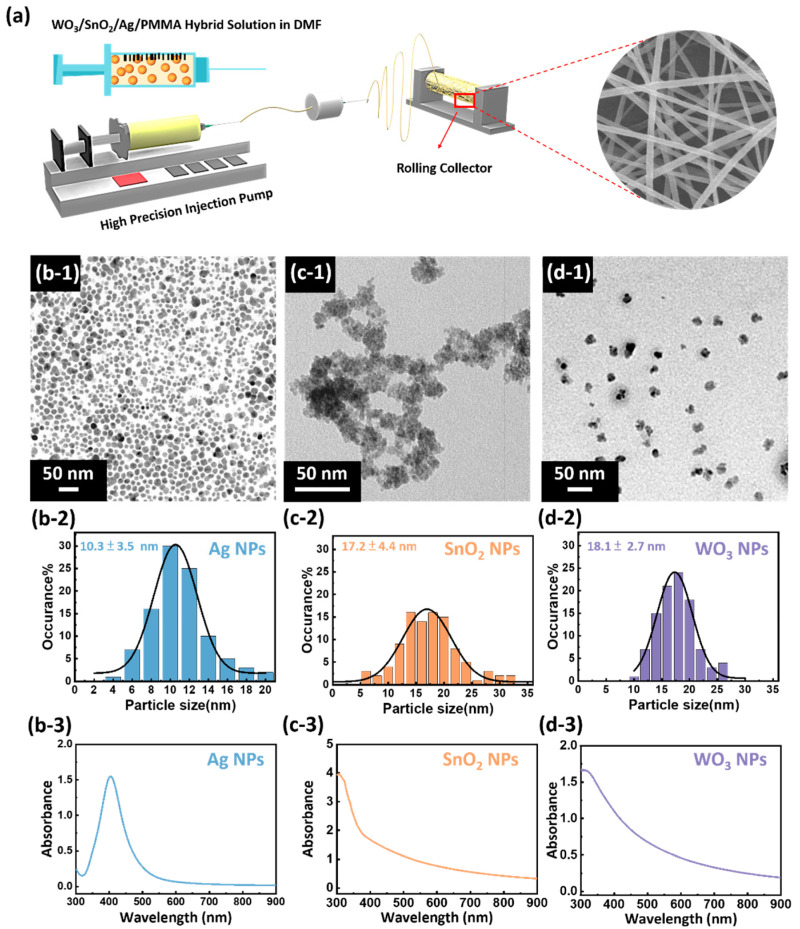
(**a**) The schematic of sensing material preparation. (**b-1**,**c-1**,**d-1**) TEM image, (**b-2**,**c-2**,**d-2**) the percentage distribution of particles sizes, and (**b-3**,**c-3**,**d-3**) UV–vis absorbance spectrum of (**b-1**,**b-2**,**b-3**) Ag NPs, (**c-1**,**c-2**,**c-3**) SnO_2_ NPs, (**d-1**,**d-2**,**d-3**) WO_3_ NPs.

**Figure 2:**
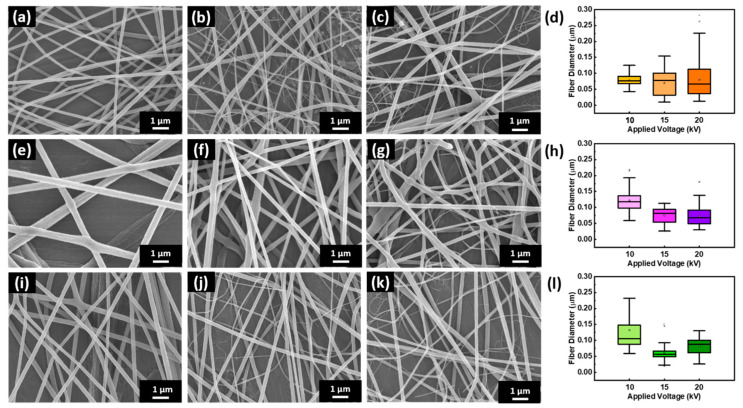
SEM images and boxplot of fiber diameter distribution of electrospun fibers: (**a**–**d**) SnO_2_/Ag/PMMA, (**e**–**h**) WO_3_/Ag/PMMA, and (**i**–**l**) WO_3_/SnO_2_/Ag/PMMA. Electric field: (**a**,**e**,**i**) 10 kV, (**b**,**f**,**j**) 15 kV, and (**i**,**j**,**k**) 20 kV. The boxplots show medians, interquartile ranges and outliers.

**Figure 3:**
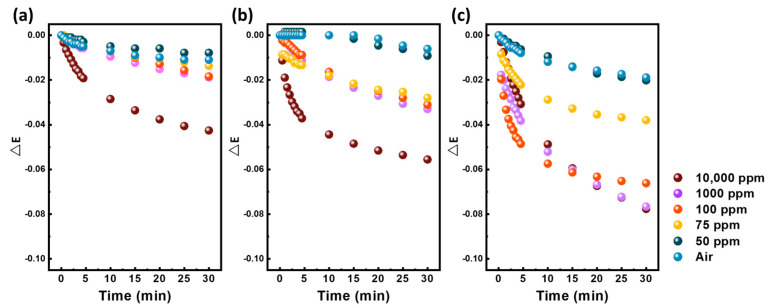
Extinction changes of (**a**) SnO_2_/Ag/PMMA fibers (**b**) WO_3_/Ag/PMMA and (**c**) WO_3_/SnO_2_/Ag/PMMA sensing materials exposed to various concentrations of acetone.

To specifically identify acetone, a diabetic breath biomarker, three nanoparticles, including Ag, SnO_2_, WO_3_, were chosen as sensing materials to enhance the acetone detection sensitivity. The schematic showing the preparation of the WO_3_/SnO_2_/Ag/PMMA sensing materials is shown in Figure 1a. The fibrous films were prepared through electrospinning technique using the WO_3_/SnO_2_/Ag/PMMA hybrid solution in DMF. The microstructures and size distribution of the synthesized Ag, SnO_2_, and WO_3_ were investigated by TEM. The TEM images exhibited uniformly mono−dispersed spherical Ag NPs. The histogram of the size distribution showed an average spherical Ag NPs size of 10 nm (Figure 1(b-1,b-2)). The UV−visible spectrum of the Ag NPs showed an absorption peak at around 430 nm (Figure 1(b-3)). Due to the surface plasmon resonance effect (SPR), Ag NPs usually absorb light noticeably and intensely at a specific wavelength between 400–450 nm. Thus, the introduction of Ag NPs into the sensing material is expected to increase the sensitivity of the gas sensing effectively. The TEM image of SnO_2_ NPs Figure 1(c-1,c-2) clearly indicates the successful synthesis of small nanodimensional spherical SnO_2_ NPs with an average diameter of 17 nm. The absorption edge of SnO_2_ NPs was located at around 390 nm (Figure 1(c-3)). Spherical WO_3_ NPs can be observed through the TEM image in Figure 1(d-1). The histogram of size distribution calculated from the TEM image shows an average diameter of 18 nm (Figure 1(d-2)). The absorption edge of WO_3_ NPs was located at around 490 nm (Figure 1(d-3)). Considering the advantages of Ag, SnO_2_, and WO_3_ NPs, the designed WO_3_/SnO_2_/Ag/PMMA sensing material is expected to enhance the sensitivity of VOCs sensing.

Both the applied voltage and working distance have been shown to have varying effects on fiber diameter. Here, SnO_2_/Ag/PMMA (Figure 2a–c), WO_3_/Ag/PMMA (Figure 2e–g) and WO_3_/SnO_2_/Ag/PMMA (Figure 2i–k) fibers with various applied voltage were deposited on glass substrates to understand the effect on the surface morphology and optical properties. An electric field is formed between the spinneret nozzle and the grounded collector by applying a voltage of 10 kV (Figure 2a,e,i), 15 kV (Figure 2b,f,j), and 20 kV (Figure 2c,g,k), respectively. The diameter distribution of nanofibers are presented in Figure 2d,h,l and summarized in Table 1. It is established that the diameter of nanofibers decreased as the voltage increased. The appropriate voltage could ensure the formation of a stable Taylor cone for uniform electrospun fibers [32,33]. Fundamentally, reducing the fiber diameter increases the specific surface area, leading to stronger VOCs absorption behavior. The smallest diameter of SnO_2_/Ag/PMMA, WO_3_/Ag/PMMA and WO_3_/SnO_2_/Ag/PMMA fibrous film was produced using an applied voltage of 20 kV. However, these nanofibers showed irregular diameter distributions, which hindered the reproducibility and reliability of the sensor. Therefore, we chose the optimal voltage—10 kV for SnO_2_/Ag/PMMA and 15 kV for WO_3_/Ag/PMMA and WO_3_/SnO_2_/Ag/PMMA—that produced the most uniformly distributed fibers for sensing material production.

Figure 3 shows the extinction change of SnO_2_/Ag/PMMA, WO_3_/Ag/PMMA and WO_3_/SnO_2_/Ag/PMMA, when exposed to various concentrations of acetone. A control experiment using water simulates the moisture in the background (i.e., air) and is used as a relative reference to avoid detection mistakes. Based on our previous work, we have found that PMMA mixing with Ag NPs can efficiently enhance the detection limit of the sensing material from surface plasmon resonances. However, the detection limit of the Ag/PMMA sensing material could only reach 100 ppm acetone vapor (Figure S1). Therefore, we further introduced SnO_2_ to increase selectivity and WO_3_ to take advantage of its gasochromic property. It can be observed that the addition of SnO_2_ actually decreased the response intensity instead of increasing it. For the WO_3_/Ag/PMMA sensing material, the detection limit was also reduced to 75 ppm. However, the WO_3_/SnO_2_/Ag/PMMA sensing material demonstrated a synergistic effect, greatly increasing the extinction change and shortening the response time. Moreover, both SnO_2_ and WO_3_ are n−type semiconductors. The interfaces between SnO_2_ and WO_3_ might construct n−n junctions, where a part of W^6+^ ions will substitute Sn^4+^ ions in SnO_2_ through n−type doping. Therefore, it would generate more electron–hole pairs than those without such structure when the light strikes [34]. This helps to improve the sensitivity of sensing materials when they are subjected to acetone surroundings.

**Figure 4:**
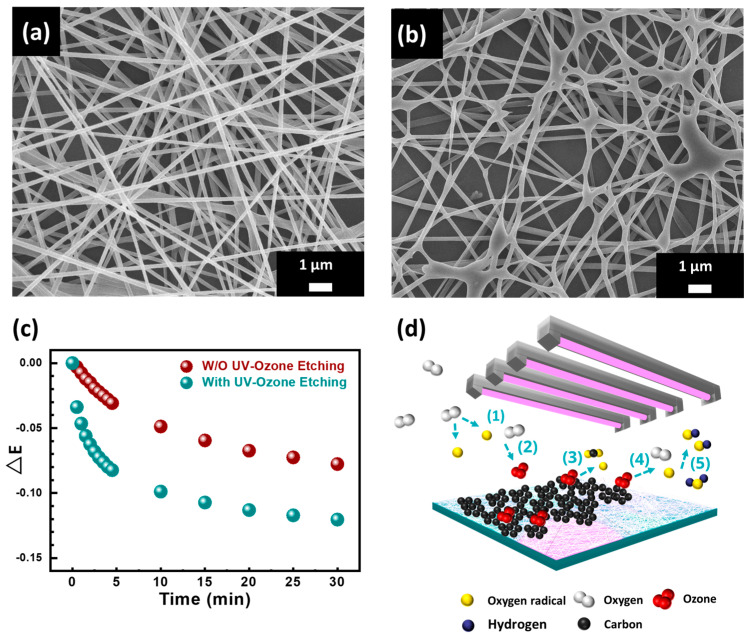
SEM images of WO_3_/SnO_2_/Ag/PMMA with UV−ozone etching: (**a**) non−etched and (**b**) 10 min etched. (**c**) Extinction changes of WO_3_/SnO_2_/Ag/PMMA sensing materials with and without etching when exposed to 10,000 ppm acetone. (**d**) Schematic diagram of UV–ozone etching process.

To increase the specific surface area of the WO_3_/SnO_2_/Ag/PMMA sensing materials, we etched the specimen’s surface with UV−ozone for 10 min. It is noteworthy to observe the morphological changes in the SEM images (Figure 4a,b). The surface area of the WO_3_/SnO_2_/Ag/PMMA sensing materials gradually eroded when etched and further enriched the specific surface area. We infer that the sensitivity of the sensing material is related to the specific surface area of the WO_3_/SnO_2_/Ag/PMMA sensing materials. To investigate the effect of UV−ozone treatment, the sensing material with and without etching were exposed to a concentration of 10,000 ppm acetone vapor for 30 min (Figure 4c). The extinction change shows a noticeable improvement after UV−ozone treatment. This is attributed to the deformation of the electrospun fibers. The etched fibers became fragile and easily reacted with VOCs. Besides, the UV–ozone is a simple and effective method for increasing the hydrophilicity of the sensing material to enhance the VOCs adsorption behavior. The schematic diagram of UV–ozone mechanism is shown in Figure 4d. Driven by the UV radiation, oxygen molecules (O_2_) dissociate and split into oxygen radicals (O·). These oxygen radicals recombine with oxygen molecules to form ozone (O_3_). At the same time, O_3_ reacts with organic molecules absorbed by the substrate to form carbon dioxide (CO_2_) and water (H_2_O), which leave the substrate during the UV–ozone treatment. Next, driven by the UV radiation, the formation of hydroxyl radicals (·OH) by ozone photolysis provides a chemically active surface for VOCs adsorption [35,36].

(1) $$O_{2}+hv\to 2O\;\cdot$$

(2) $$O\;\cdot +\;O_{2}\to \;O_{3}$$

(3) $$O_{3}\;+\;organic\;molecule\to CO_{2}+H_{2}O$$

(4) $$O_{3}\;+hv\to O\;\cdot +\;O_{2}$$

(5) $$O\;\cdot +H_{2}O\to 2OH\;\cdot$$

**Figure 5:**
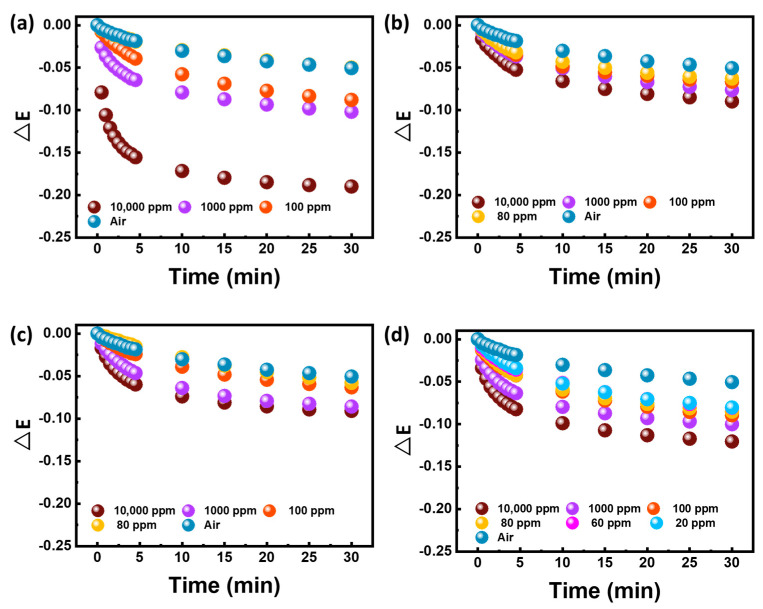
Extinction changes of the WO_3_/SnO_2_/Ag/PMMA sensing materials exposed to various concentrations of (**a**) ethanol, (**b**) p−Xylene, (**c**) toluene, and (**d**) acetone.

The WO_3_/SnO_2_/Ag/PMMA sensing material was successfully prepared by adjusting the fiber diameter and UV–ozone etching. The surface area of the sensing material can be effectively increased from the surface treatment, showing a good adsorption effect on VOCs. We suggest that the etched fibers were too fragile to maintain their shape, resulting in a shortened response time. In addition to detecting the acetone biomarker, we also detected other common VOCs in the industry, ethanol, p−Xylene, and toluene. The extinction spectra and response time are summarized in Figure 5 and Table 2. The developed sensing material can detect 20 ppm acetone vapor within 10 min. Toluene and xylene can both be effectively sensed at 80 ppm. The result from ethanol showed that the detection limit of ethanol can reach 100 ppm. The conversion of a hydrophobic surface to a hydrophilic surface after UV–ozone treatment can have several effects on the behavior of chemical species that interact with that material surface. In the case of toluene and p−Xylene, which are both hydrophobic, the hydrophilic surface generated by UV−ozone treatment could potentially reduce their affinity for the sensor surface. The change in surface chemistry induced by the UV−ozone treatment may also affect the kinetics of adsorption and desorption of toluene and p−Xylene on the sensor surface. Hydrophilic surfaces typically exhibit faster adsorption rates for polar or charged molecules, which could lead to shorter response times for these species, such as acetone and ethanol, in a sensor application. Figure S2 illustrates the error bars chart of the extinction change of the WO_3_/SnO_2_/Ag/PMMA sensing material under each exposing concentration, calculated through three tests. Few deviations were observed in the extinction change curve during the detection of acetone at concentrations of 10,000, 1000, and 100 ppm. This indicates the high reliability of WO_3_/SnO_2_/Ag/PMMA sensing materials when using the developed technique. In addition, the as−fabricated acetone sensor performances in this paper were compared with that of other sensors, as shown in Table 3. Most of the sensors, such as conductometric and chemiresistive sensors, which perform optimally under high temperatures and are susceptible to humidity, can reach a ppb level detection. Exhaled breath preconcentration is a procedure in which exhaled breath samples are concentrated to increase the sensitivity of subsequent analyses. For improving the detection performance, preconcentration could be a way to improve the detection limit of analytical instruments by increasing the concentration of the target analytes and reducing the interference from other components in the breath sample. The WO_3_/SnO_2_/Ag/PMMA sensing material still demonstrates unique performance, including high sensitivity, low−cost, and operation at room temperature.

**Figure 6:**
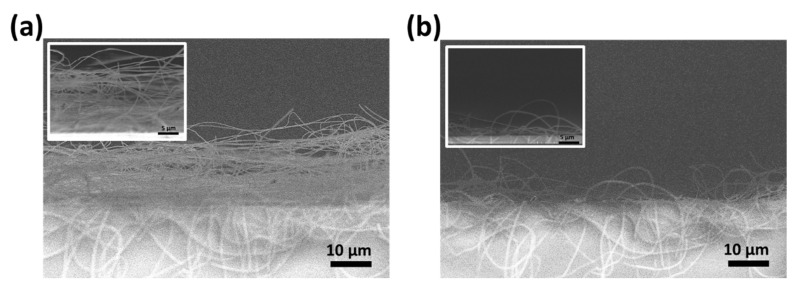
The cross−sectional FESEM images of WO_3_/SnO_2_/Ag/PMMA sensing materials (**a**) before and (**b**) after being exposed to acetone.

**Figure 7:**
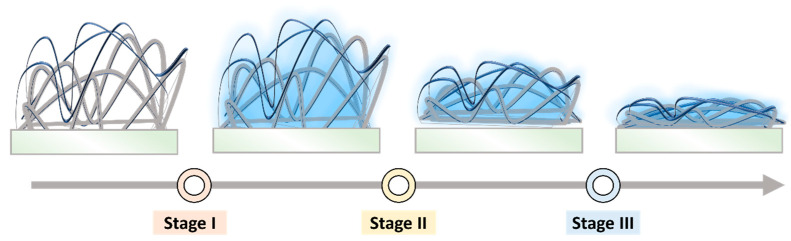
Schematic diagram of the VOC sensing behavior.

In order to verify the nanostructures of our sensing material and establish the sensing mechanism, FESEM was carried out. Figure 6 demonstrates the cross−sectional FESEM images of WO_3_/SnO_2_/Ag/PMMA sensing materials before and after exposure to acetone. Prior to exposure, the electrospun fiber gave the surface a 3D structure. After exposure to VOC environment, the fiber exhibited obvious collapse behavior. Based on our observations, we developed a hypothesis for the sensing mechanism of the material. For the developed sensing material, the sensing behavior can be divided into three stages as illustrated in Figure 7. In stage I, when the sensing material is exposed to VOCs in the surroundings, the hydrophilic surface of the sensing material readily adsorbs the VOCs present in the environment onto the 3D fibrous film. This is due to the increased affinity of the hydrophilic surface to acetone after UV−ozone treatment. In the stage II, the adsorbed VOCs cause polymer relaxation or swelling of the fibrous film, which leads to the collapse of the fibrous structure. This collapse might be due to changes in the intermolecular forces between the polymer chains, which can result from the presence of the adsorbed VOCs. Finally, the fibers of the sensing material experience over−swelling, which results in the complete collapse of the 3D fibrous structure. This collapse causes the disappearance of the 3D scattering effect, which is a key feature of the sensing material’s response to VOCs.

## 4. Conclusions

WO_3_/SnO_2_/Ag/PMMA sensing materials were successfully synthesized by the electrospinning technique. Signal enhancement by Ag NPs can enhance the sensitivity of the developed sensing materials. Besides, by constructing the heterostructure of SnO_2_ and WO_3_, we can utilize their selectivity and gasochromic property, respectively. The interfaces between SnO_2_ and WO_3_ might construct n−n junctions, which generate more electron–hole pairs than those without such structure when the light strikes. This helps to improve the sensitivity of sensing materials when they are subjected to acetone surroundings. The specific surface area was successfully increased after the UV−ozone treatment. The etched fibers were too fragile to maintain their shape and easily reacted with VOCs, resulting in increased sensitivity and a shortened response time. The developed WO_3_/SnO_2_/Ag/PMMA sensing material can detect 20 ppm acetone vapor within 10 min under room temperature. It is hoped that the developed sensing material can have a great impact on the development of sensors to reduce the frequency and stress of invasive testing for diabetic patients.

## Figures and Tables

**Table 1:** Average diameter of SnO_2_/Ag/PMMA, WO_3_/Ag/PMMA and WO_3_/SnO_2_/Ag/PMMA fibers with various applied voltage.

Sample	Applied Voltage (kV)	Fiber Diameter (nm)
SnO_2_/Ag/PMMA	10	78 ± 16
	15	69 ± 38
	20	80 ±54
WO_3_/Ag/PMMA	10	122 ± 32
	15	75 ± 23
	20	72 ± 30
WO_3_/SnO_2_/Ag/PMMA	10	132 ± 68
	15	61 ± 22
	20	82 ± 26

**Table 2:** Detection limit and response time of the WO_3_/SnO_2_/Ag/PMMA sensing materials when exposed to various VOCs concentrations.

VOCs	Detection Limit (ppm)	△E	Responses Time (min)
Ethanol	100	−0.087	10
p−Xylene	80	−0.061	20
Toluene	80	−0.058	25
Acetone	20	−0.081	10

**Table 3:** Acetone sensing properties of with various types of reported sensing materials.

Materials	Sensor Type	Detection Limit	Detection Time	Operation Temp. (°C)	Ref.
WO_3_−In_2_O_3_	conductometric	0.1 ppm	real−time	280	[37]
SnSe_2_/SnO_2_	conductometric	0.354 ppm	real−time	300	[38]
γ−WO_3_	chemiresistive	0.2 ppm	20 s	300	[39]
N−doped carbon dots	colorimetric	0.5 mM	−	room temperature	[40]
Co_3_O_4_^−^TiO_2_	conductometric	0.1 ppm	122 s	250	[41]
Ru−Co_3_O_4_	conductometric	50 ppb	real−time	137.5	[42]

## Data Availability

The data presented in this study are available on request from the corresponding author.

## Supplementary Materials

The following supporting information can be downloaded at: https://www.mdpi.com/article/10.3390/polym15081833/s1, Figure S1: Extinction changes of Ag/PMMA fibers sensing materials when exposed to various concentrations of acetone; Figure S2: Repeatability of the WO_3_/SnO_2_/Ag/PMMA sensing materials exposed to acetone with various concentration.
