# Supplementary material for: Electrospun Fibrous Nanocomposite Sensing Materials for Monitoring Biomarkers in Exhaled Breath
Source: Polymers (Basel). 2023 Apr 10;15(8):1833. doi: 10.3390/polym15081833 (PMC10143897; doi:10.3390/polym15081833)
Supplement: Supplementary file 1 [file polymers-15-01833-s001.zip › polymers-2247279-supplementary.pdf]

Supporting Information

Electrospun Fibrous Nanocomposite Sensing Materials for Monitoring Biomarkers in Exhaled Breath

Yin-Hsuan Chang [1], Ting-Hung Hsieh [1], Kai-Chi Hsiao [1], Ting-Han Lin [1], Kai-Hsiang Hsu [2,*] and Ming-Chung Wu [1,2,3,*]

[1] Department of Chemical and Materials Engineering, Chang Gung University, Taoyuan 33302, Taiwan

[2] Division of Pediatric Neonatology, Department of Pediatrics, Chang Gung Memorial Hospital, Linkou, Taoyuan 33305, Taiwan

[3] Green Technology Research Center, Chang Gung University, Taoyuan 33302, Taiwan

* Correspondence: khsu@cgmh.org.tw (K.-H.H.); mingchungwu@cgu.edu.tw (M.-C.W.)

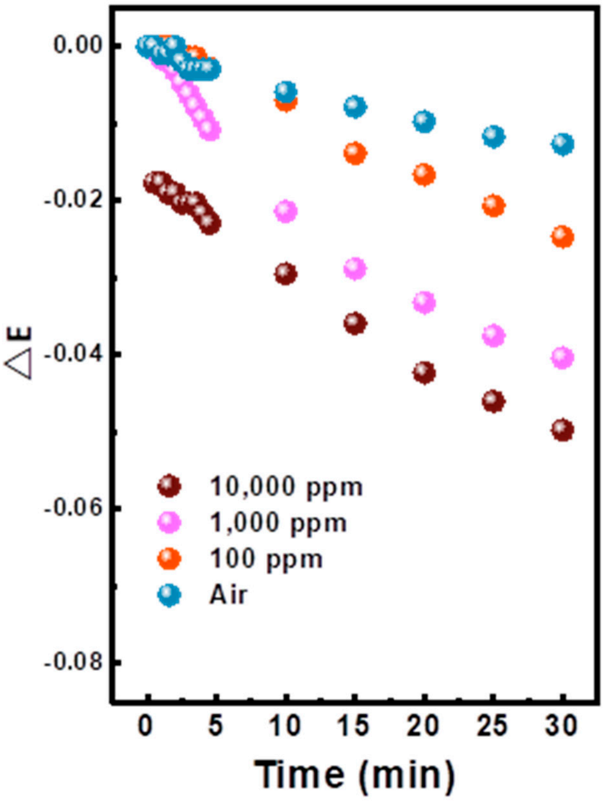

Figure S1 Extinction changes of Ag/PMMA fibers sensing materials when exposed to various concentrations of acetone.

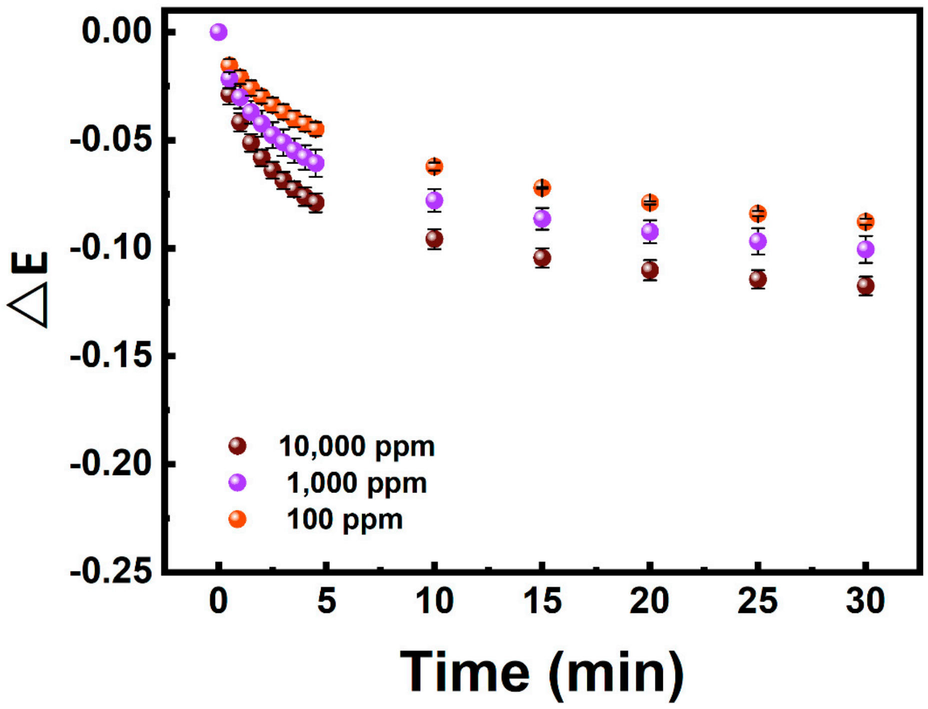

Figure S2. Repeatability of the WO_3/SnO_2/Ag/PMMA sensing materials exposed to acetone with various concentration.
